# The Role of Lifestyle on Adherence to Treatment in a Sample of Patients with Unipolar and Bipolar Depression

**DOI:** 10.3390/ijerph20031994

**Published:** 2023-01-21

**Authors:** Beatrice Benatti, Nicolaja Girone, Dario Conti, Maddalena Cocchi, Francesco Achilli, Silvia Leo, Gianmarco Putti, Monica Bosi, Bernardo Dell’Osso

**Affiliations:** 1 Department of Mental Health, Department of Biomedical and Clinical Sciences Luigi Sacco, University of Milan, 20122 Milan, Italy; 2 “Aldo Ravelli” Center for Neurotechnology and Brain Therapeutic, University of Milan, 20122 Milan, Italy; 3 Department of Psychiatry and Behavioral Sciences, Bipolar Disorders Clinic, Stanford University, Stanford, CA 94305, USA

**Keywords:** depression, adherence to treatment, lifestyle

## Abstract

**Introduction:** Poor adherence to treatment is currently stated to be one of the causes of depression relapse and recurrence. The aim of the present study is to assess potential differences in terms of clinical and lifestyle features related to adherence to treatment in a sample of patients with unipolar and bipolar depression. **Methods:** One hundred and eight patients with a diagnosis of unipolar or bipolar depressive episode were recruited from January 2021 to October 2022. Adherence to psychopharmacological treatment was assessed using the clinician rating scale. Descriptive and association analyses were performed to compare subgroups based on adherence to treatment. **Results:** Lower levels of adherence to treatment were associated with fewer years of education, work impairment, manic prevalent polarity lifetime, and greater comorbidity with alcohol and drug abuse. The majority of patients with positive adherence did not report any hospitalization and involuntary commitment lifetime. **Conclusions:** Patients with a positive treatment adherence showed significant differences in terms of lifestyle and clinical features compared to non-adherent patients. Our results may help to identify patients more likely to have poor medication adherence, which seem to lead to a worse disease course and quality of life.

## 1. Introduction

Depression represents one of the leading causes of disability worldwide [1], with an estimated prevalence of about 5% of the global population, resulting from a complex interaction of social, psychological, and biological events. Interrelationships between depression and physical health have been recognized [2]. Major depression (MD) frequently recurs in non-clinical cohorts; one-third of people who have experienced at least one episode will have another [3]. The average number of episodes per patient is four, with a mean length of 14–17 weeks for moderate episodes and 23 weeks for severe episodes [4].

(a) Depression and medical comorbidities

All the different forms of depression are common in the context of medical setting and even the less severe forms may become disabling and impact the course of the primary medical illness [5]. Among the most common comorbidities, conditions such as chronic kidney disease (20–50%), cancer (15–25%), coronary artery disease (15–23%), and diabetes (12–18%) can be found and may also be considered an independent risk factor for the development of depression [6]. It is also worth mentioning that depression is thought to contribute to poor glycemic control, due to both poor adherence to diet/medication regimen and metabolic effects of increased stress state and cortisol release [7]. Other common comorbidities are multiple sclerosis (MS) with a lifetime depression prevalence ranging from 23% to 54% [5] and chronic pain, with more than a half of patients manifesting both the conditions. The most common chronic pain conditions associated with depression are fibromyalgia, chronic headache, and chronic pelvic pain [6,7,8]. The role of interoception underlying such conditions of comorbidity is currently under investigation. In this perspective, major depression proved to be, in some cases, associated with vagus nerve, insula, and anterior cingulate circuit dysfunctions, which all have a potential role in interoception [9].

(b) Depression and substance use

Epidemiological studies have found that the co-occurrence of substance use disorders (SUDs) and other psychiatric disorders are relatively common in the general populations in countries where it has been investigated (e.g., Europe, Australia, New Zealand, and North America). The Substance Abuse and Mental Health Services Administration [10] released estimates from the 2012 National Survey on Drug Use and Health (NSDUH), indicating that among U.S. adults with a past year SUD, 40.7% had a co-occurring mental health disorder and these same adults accounted for 19.2% individuals over 18 who had any past-year mental health disorder and also met criteria for SUD [11]. MD has extremely high rates of comorbidity with SUD. On the other hand, MD may induce SUDs, SUD may contribute to MD development, or underlying vulnerabilities and common life experience may confer risk to develop both conditions [12]. A meta-analysis conducted on epidemiological surveys between 1990 and 2014 revealed the strongest associations between illicit drug use disorder and major depression (pooled OR 3.80, 95% CI 3.02-4.78) and alcohol use disorders and major depression (OR 2.42, 95% CI 2.22–2.64) [13]. More in detail, depressive disorders are the most common psychiatric disorders among people with alcohol use disorder (AUD). The co-occurrence of these disorders is associated with greater severity and worse prognosis than either disorder alone, including a heightened risk for suicidal behavior [14]. As regards gender prevalence, studies performed both in the general population and in the clinical population showed that the comorbidity of MD and SUD are more frequent in women than in men in the clinical context, and it is twice as frequent in women from non-clinical samples. Thus, women with SUD constitute a particularly vulnerable group [15]. Co-occurring depression has an adverse effect on the course of SUDs. In fact, a current depressive episode may predict a poorer treatment response and higher rates of relapse. Similarly, the effects of SUDs on the course of MD have been reported. The National Longitudinal Alcohol Epidemiological Survey found that past alcohol dependence significantly increased the risk of past-year MD [16]. Conversely, an epidemiological survey found that among people with lifetime SUDs and MD, a past-year substance dependence remission was associated with a reduced risk of depression [17].

(c) Depression and poor adherence to treatment

MD treatment availability is constantly increasing and improving, new drugs are better tolerated than their predecessors and significantly improve patient adherence [18]. Nevertheless, poor adherence to treatment is currently stated to be one of the causes of depression relapse and recurrence [19,20]. In particular, treatment non-adherence has been associated with a worse outcome, such as lower remission rates [21] and less syndromal recovery [22]. Recurrence of affective episodes was associated with cumulative increases of morbidity risks [23,24], treatment nonresponse [25], full syndromal recurrence [26,27], and suicidality [28]. Furthermore, a study found that adopting measures to improve good treatment adherence may reduce the frequency of relapse in patients with recurrent or chronic depression [29].

Adherence to treatment is a common topic across every medical field, especially when considering chronic diseases, and potential predictors have been deeply studied. Factors that may predict treatment adherence are physician communication [30] and information given by healthcare personnel every time they see the patient [31], interpretation of illness and wellness [32], social support [33], scholastic education and employment [34], side effects [35], SUD [36]. Depression and anxiety are themselves negative predictors for good therapeutic compliance [37]; thus, it is self-evident that people suffering from depression are at risk for relapse and recurrence as much as, if not more, the other medical morbidities.

The aim and novelty of the present study was to assess potential differences in terms of clinical and socio-demographic characteristics related to lifestyle such as adherence to treatment, medical comorbidities, and substance use in a sample of patients diagnosed with unipolar and bipolar depression in different clinical settings of an Italian psychiatric department.

## 2. Methods

### 2.1. Sample

One hundred and eight patients with a DSM-5 diagnosis of unipolar or bipolar depressive episode, of either gender or any age (69.4% female rate and a mean age of 50.3 ± 16.2 years) were recruited from the different psychiatric services (one tertiary clinic, one day-hospital service, two community mental health centers, one psychiatric ward, and one high-assistance rehabilitation community) of the Department of Psychiatry of Luigi Sacco University Hospital in Milan. Diagnoses were assessed by means of a semi-structured interview based on DSM-5 criteria (SCID-5) [38]. Moreover, a set of psychometric measures were administered: Hamilton Depression Rating Scale (HDRS-21) [39], Hamilton Anxiety Rating Scale HAM-A [40], Montgomery Asberg Depression Rating Scale (MADRS) [41], Montreal Cognitive Assessment (MoCa) [42], and Clinician Rating Scale (CRS) [43,44]. In particular, the HDRS-21 is a 21-item clinician-administered scale that measures the severity of depression, with a special focus on somatic symptoms; the HAM-A is a widely used 14-item clinician-administered rating tool measuring the severity of anxiety symptoms; the MADRS is a 10-item, clinician-administered scale aiming to assess core mood symptoms of depression; the CRS uses an ordinal scale to quantify the clinician’s assessment of the level of adherence shown by the patient; and MoCA was designed as a rapid screening instrument for mild cognitive dysfunction.

Inclusion criteria were: (a) a DSM-5 diagnosis of unipolar or bipolar depressive episode; (b) age > 18 years at the time of inclusion; and (c) the presence of written informed consent. Exclusion criteria will comprise: (a) incapacity of giving informed consent; and (b) intellectual disability. There were no exclusion criteria in relation to substance abuse, concomitant/previous pharmacological treatments, legal status, or medical comorbidity.

Data collection took place from January 2021 to October 2022. All medical records of recruited patients were retrospectively reviewed, anonymized, and held in a secure database according to the local data protection policies. Patients gave their informed consent to participate in this study and to have their personal, clinical, and demographic data used for research purposes. The present study was conducted according to the ethical standards of the relevant national and institutional committees on human experimentation and the principles expressed in the Declaration of Helsinki (PMC2566407). The patients provided their written informed consent to participate in this study and for the use of their anonymized data for research purposes.

### 2.2. Outcome Measures

Main clinical and socio-demographic variables were collected by reviewing patients’ medical records. Specific attention was paid to present/past history of substance and alcohol abuse, and history of medical comorbidities such as obesity, hypertension, diabetes, and high blood cholesterol levels. Moreover, adherence to psychopharmacological treatment was assessed using the CRS. The scale is interviewer-administered and based on an ordinal scoring from 1 to 7 to quantify the level of adherence shown by the patient. Higher scores represent greater patient adherence to pharmacological treatment. According to the CRS score, for the purpose of the study, the whole sample was divided into two subgroups based on adherence to treatment (A+: positive adherence to treatment; A−: negative adherence to treatment).

For the purpose of the present study, selected analyzed variables included: age; gender; service; age at onset; educational status; years of education; marital status; professional status; living alone; duration of illness (months); duration of untreated illness (DUI, months); positive family history for psychiatric disorder; diagnosis; prevalent polarity (one polarity occurring during at least two-third of lifetime episodes, i.e., depressive predominant polarity or manic/hypomanic predominant polarity) [45]; lifetime presence of comorbid psychiatric disorders and type; lifetime medical comorbidities and type; current substance use; number of hospitalizations lifetime; number of involuntary commitments lifetime; psychotherapy lifetime; CRS score; psychometric scales scores; adherence to psychopharmacological treatment (A+: positive adherence to treatment; A−: negative adherence to treatment).

### 2.3. Statistical Analyses

Patient socio-demographic and clinical characteristics are presented using descriptive statistics. Student’s *t*-test and one-way ANOVA for continuous variables and χ^2^ test for dichotomous ones were performed for comparison of sociodemographic and clinical features between adherence to treatment subgroups. Next, the bivariate correlation between continuous variables of the study was estimated using Pearson’s correlation for the whole sample. Multivariate logistic regressions were performed to analyze possible factors associated with adherence to treatment.

A *p*-value < 0.05 was considered statistically significant. Statistical analyses were performed using IBM SPSS Statistics V26.0 (IBM Corp, Armonk, NY, USA).

## 3. Results

**Figure 1:**
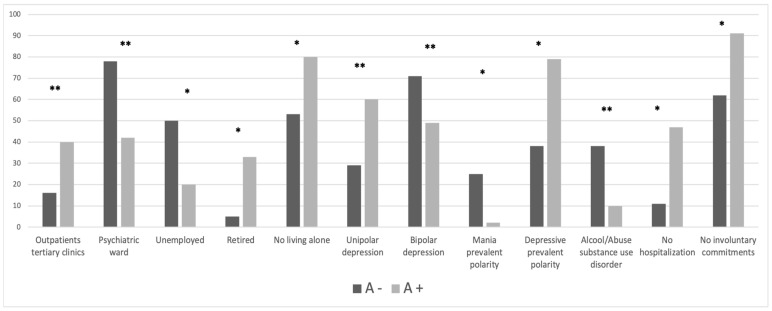
Comparison of sociodemographic and clinical features between subgroups. **Notes:** A−: negative adherence to treatment, A+: positive adherence to treatment. ** *p* < 0.005 * *p* < 0.05.

The sample included 108 patients with a diagnosis of unipolar MD episode (51%) and bipolar MD Episode (49%), distributed as follows: 42% patients from outpatients tertiary clinic, 53.3% from psychiatric ward, and 6.7% from community mental health center. The main socio-demographic and clinical variables of the study sample are provided in Table 1 and Figure 1.

The whole sample showed a 69.4% female rate and a mean age of 50.3 ± 16.2 years. Regarding clinical features, the mean age at mood disorder onset was 29.9 ± 12.5 years with a mean duration of illness of 232.1 ± 156.6 months and a mean duration of untreated illness of 28.4 ± 45.3 months. The majority of the sample reported a prevalent depressive polarity lifetime (68.3%). Seventy percent of the sample reported a positive psychiatric family history for mood and other psychiatric disorders. Moreover, 54.4% of the sample showed psychiatric comorbidity, with SUD as the most represented (16.2%), followed by anxiety disorders (14.7%). At the time of inclusion in the study, 6.9% of patients described current substance use. Fifty-nine percent of the whole sample reported a lifetime medical comorbidity, hypertension being the most represented (19%). Moreover, 25% of the sample showed at least one psychiatric hospitalization lifetime, and 11.4% at least one involuntary commitment lifetime. As regards pharmacological treatment at the time of inclusion in the study, the majority of the sample (92.9%) was assuming a polytherapy, i.e., a combination of one or more antidepressants, mood stabilizers (lithium, antiepileptics), and/or antipsychotics.

For the purposes of the study, the total sample was divided into two subgroups based on adherence to pharmacological treatment.

Significantly higher rates of inpatients from psychiatric ward were A− compared to A+ patients (78.1% vs. 42.5%, *p* < 0.005); the majority of A- patients were actually recruited from the inpatient ward compared to other psychiatric services. A− patients were significantly more unemployed (50% vs. 20%, *p* < 0.05), were mostly living in their family of origin (47.6% vs. 20%, *p* < 0.05), and had fewer years of education compared to A+ subgroup (10.63 ± 3.18 vs. 12.3 ± 3.2 years, *p* < 0.05). Moreover, lower rates of retired status emerged in the A− subgroup (4.5% vs. 33.3%, *p* < 0.05).

Higher rates of bipolar depression (BD) diagnosis and a prevalent manic polarity lifetime emerged in A− compared to the A+ group (71% vs. 39.7%, *p* < 0.005; 25% vs. 2.1%, *p* < 0.05, respectively). Moreover, A+ reported significantly higher rates of depressive prevalent polarity lifetime (78.7% vs. 37.5%, *p* < 0.05).

With regards to clinical status, A− patients reported significantly higher rates of comorbidity with alcohol use or other SUD lifetime (37.5% vs. 9.6%, *p* < 0.005). The majority of A+ patients did not report any psychiatric hospitalization and involuntary commitment lifetime (46.3% vs. 11.1%, *p* < 0.05; 90.7% vs. 62.5%, *p* < 0.05, respectively).

Furthermore, though not reaching a significant difference compared to A− group, A+ patients showed higher rates of presence of medical comorbidities lifetime (66% vs. 42.1%, *p* = 0.068; see Table 1).

With regard to psychometric questionnaires, significantly higher scores of HAM-A were observed in A+ groups compared to A− (12.9 ± 18.3 vs. 5.63 ± 13.6, *p* < 0.005). No other differences emerged for the other psychometric measures (see Table 2).

Finally, when bivariate correlation was performed, a positive correlation between years of education and scores of adherence to pharmacological treatment rates by CRS scale emerged for the whole sample (r = 0.229, *p* = 0.044). No other significant correlations emerged. Moreover, no predictive effect of specific clinical and sociodemographic factors was found for adherence to treatment.

## 4. Discussion

In the present study we analyzed the possible socio-demographic and clinical factors related to the levels of therapeutic adherence in a sample of patients with mood disorders in different mental health services of an Italian psychiatric department.

The first finding of the study was that higher rates of non-adherent subjects were found in psychiatric acute wards compared to other psychiatric services. These results are consistent with previous research. More in detail, a systematic review by Ho and colleagues showed that patients with unipolar depression who were non-adherent were at increased risk of relapse and recurrence, and showed increased rates of psychiatric hospitalization [46]. Another study reported that BD subjects with a current inpatient status were more at risk of being non-adherent [28].

One-third of the whole sample reported having suboptimal medication adherence. Poor adherence to medication in people with MD has been widely reported in the literature; however, the rates of non-adherence vary considerably across studies (30–97%) [47,48,49]. In this sense, our results are congruent with, but ranking at the lower end of, the previous reported rates. This can be explained considering that the study sample was composed of depressed and bipolar patients recruited mostly during an ongoing pharmacological treatment in a tertiary center highly specialized in mood disorders. Thus, this is probably a selected population particularly adherent to treatment and mildly to moderately ill, as reported by the mean scores of the psychometric questionnaires (HDRS-21: 13.7 ± 18.8; MADRS: 18.3 ± 25.7).

The analysis of socio-demographic characteristics showed fewer years of education in the A− group compared to A+ group. Moreover, a positive correlation between years of education and adherence to pharmacological treatment scores emerged for the whole sample. Previous research has described a lower level of education as a risk factor for non-adherence to medication and psychoeducation groups [28,50]. A large study of 489 subjects with BD, conducted by Johnson and colleagues, reported that patients with a higher education were more likely to be adherent to pharmacological treatments [51]. On the basis of these findings, patients whose educational level is lower may be more likely to be non-adherent and extra effort should be made to ensure that they fully understand the instructions, the expected time for clinical improvement, the possible side effects, and the importance of regular dosing. However, some of the literature showed mixed findings [52].

Work impairment has been linked to non-adherence in our study; a significantly higher unemployment status emerged in the A− group compared to A+, in line with the current literature. Prior research showed that depressed patients who were unemployed reported significantly lower adherence to treatments than participants who were currently working [53]. On the other hand, depressive disorders predicted suboptimal adherence to treatment, substantial losses in work performance, and increased risk of unemployment [54,55,56]. Thus, effective treatment through better adherence to antidepressant drug therapies can substantially reduce the overall costs associated with MD [57]. However, some studies did not find an association between the rate of medication adherence and employment in patients with depressive disorder [58].

Also, A− patients were more frequently living in their family of origin compared to A+ subgroup. As reported in the current literature, medication nonadherence has been associated with low education and income [59,60]. Thus, our findings may be related to the inability of A− patients to afford a house on their own and the need to rely on caregivers’ help.

Moreover, from the analysis of the clinical characteristics of the sample, the A− group showed significantly higher rates of BD patients compared to the A+ group. More in detail, among BD patients, those with a lifetime manic polarity were significantly more represented in the A− group compared to the A+ group. Contrariwise, higher rates of lifetime depressive polarity emerged in BD patients of the A+ group. A study by Gonzalez-Pinto and colleagues pointed out that having more breakthrough episodes (especially manic and mixed states) in BD was related to pharmacological treatment non-adherence [61]. Generally, greater affective morbidity is related to non-adherence [62]. In addition, BD I diagnosis has been previously associated with higher rates of medication drop-out [63].

According to previous studies, we observed significantly higher rates of comorbidity with AUD and SUD lifetime in group A− compared to group A+. A research by Pacchiarotti and colleagues (2009) showed that patients with a SUD preceding the MD onset were less adherent to treatment, even though they still have a better outcome [64]. In the literature, a comorbid use of alcohol and/or drugs (especially cannabis) is one of the most strongly associated factors with nonadherence to medication in BD patients [28,62,65,66]. Moreover, co-occurring substance use in general complicates the treatment of BD and has been associated with poor adherence in other recent studies [61,67]. As regards psychometric scores, in our sample patients from the A+ group showed significantly higher HAM-A scores compared to A− group, though the prevalence of comorbid anxiety disorders were similar in the two subgroups. This is partially in contrast with the current literature: anxiety, independently or in combination with depression, has been associated with the fear of developing adverse drug reactions thus leading to non-adherence [68]; however, anxiety symptoms have also been associated with increased use of health care services and access to psychiatric treatments [37] and this could represent a possible explanation of our results. Furthermore, in our study, only A− group patients have had at least one involuntary commitment lifetime, whereas the majority of A+ patients did not report any psychiatric hospitalization. Prior studies observed that subjects with a current inpatient status are more at risk to be nonadherent [61]. Also, those patients hospitalized within the last 12 months for suicide attempts had a higher risk of being non-adherent [28].

In our study, we observed that seventy percent of the sample reported a positive psychiatric family history for mood and other psychiatric disorders. Having a strong family history of BD and suicide was related to being adherent to a psychoeducation program [69]. Possibly, a family history of BD or suicide may contribute to understand the importance of the treatment and therefore raise the motivation to treatment engagement. However, other findings showed that a positive family psychiatric history was related to drug discontinuation [70], which may be the result of long-time self-stigma [71].

Considering rates of medical comorbidities lifetime, no significant difference between adherence groups was found. Interestingly, both A− and A+ groups showed at least 40% of patients with medical comorbidities, confirming the relationship between poor lifestyle habits such as unhealthy diet, low physical activity, sedentary behaviors, and depressive episodes consistently with the current literature. [72].

No other sociodemographic factor was found to be linked to suboptimal adherence in this study. In this respect, the literature showed mixed findings on the effect of patient socioeconomic characteristics on adherence. A systematic review by Riviero-Santana reported no substantial evidence concerning the relationship between socio-demographics such as age, gender, race, educational and socioeconomic level, and adherence to pharmacological treatments [50]. On the contrary, different studies reported that medication non-adherence was consistently associated with patients’ socio-demographic characteristics (such as educational status, age, gender, and employment) [45,49,51].

The results of this work should be considered in light of some methodological limitations. First, the cross-sectional design allows only a one-time assessment and precludes any inference about the directionality of relationships. The characteristics of the sample (recruited at different stages of the treatment course) limit the comparison and generalization of results. Moreover, some variables such as psychiatric family history and age at psychiatric comorbidity onset were obtained retrospectively, being susceptible to recall bias. The use of self-reports for most of the variables is another limit, especially for the assessment of medication adherence. The limited sample size of the sample may have also influenced the study results.

## 5. Conclusions

The present study showed significant different drivers of suboptimal adherence in terms of clinical and lifestyle related features in unipolar and bipolar depressive episodes. In particular, low levels of medication adherence have been associated with reduced years of education, work impairment, higher hospitalization rates, involuntary commitments, and greater comorbidity with alcohol or drugs use. Therefore present results may help professionists to identify patients more likely to have poor medication adherence, which frequently leads to a worse disease course and quality of life. From this perspective, when treating patients with the above-mentioned features, clinicians should take specific actions such as simplifying complex medication regimens as much as possible and prefer once-daily vs. twice (or more)-daily formulations.

Further studies with larger samples are warranted to confirm present results.

## Figures and Tables

**Table 1:** Comparison of sociodemographic and clinical features between subgroups.

Variables	A−	A+	Total Sample
	n = 33 (30.6%)	n = 75 (69.4%)	n = 108
**Age**	48.8 ± 13.8	50.9 ± 17.3	50.3 ± 16.2
**Gender** (male; female)	10 (30.3%); 23 (69.7%)	23 (30.7%); 52 (69.3%)	33 (30.6%); 75 (69.4%)
**Service**			
Outpatients tertiary clinics	5 (15.6%) **	**37 (40.7%)**	42 (40%)
Psychiatric ward	25 (78.1%) **	**31 (42.5%)**	56 (53.3%)
Community mental health center	1 (6.2%)	5 (6.9%)	2 (6.7%)
**Age at onset** (years)	25.7 ± 8.4	31.3 ± 13.4	29.9 ± 12.5
**Education**			
Primary school	2 (8.3%)	4 (6.7%)	6 (7.1%)
Secondary school	7 (29.2%)	10 (16.7%)	17 (20.2%)
High school	14 (58.3%)	36 (60%)	50 (59.5%)
University	1 (4.2%)	10 (16.7%)	11 (13.1%)
**Years of education**	10.63 ± 3.18 *	**12.3 ± 3.2**	11.82 ± 3.32
**Marital status**			
Single/Separated/Divorced	15 (65.2%)	27 (45%)	42 (50.6%)
Married/Engaged	8 (34.8%)	33 (55%)	41 (49.4%)
**Professional status**			
Unemployed	11 (50%) *	**12 (20%)**	23 (28%)
Employed	10 (45.5%)	28 (46.7%)	38 (46.3%)
Retired	1 (4.5%) *	**20 (33.3%)**	21 (25.6%)
**Living alone** (yes/no)	10 (47.6%); 11 (52.4%) *	**12 (20%); 48 (80%)**	22 (27.2%); 59 (72.8%)
**Duration of illness** (months)	250.4 ± 124.3	226.8 ± 165.5	232.1 ± 156.6
**DUI** (months)	31.7 ± 49.8	21.4 ± 44.4	28.4 ± 45.3
**Positive Family history** (yes/no)	12 (57.1%); 9 (42.9%)	44 (74.6%); 15 (25.4%)	56 (70%); 24 (30%)
**Diagnosis**			
Unipolar depression	9 (29%) **	**44 (60.3%)**	53 (51%)
Bipolar depression	22 (71%) **	**29 (39.7%)**	51 (49%)
**Prevalent polarity**			
Mania	4 (25%) *	**1 (2.1%)**	5 (3.2%)
Hypomania	3 (18.8%) *	**1 (2.1%)**	4 (6.3%)
Depressive	6 (37.5%) *	**37 (78.7%)**	43 (68.3%)
Mania with mixed features	2 (12.5%)	3 (6.4%)	5 (7.9%)
Hypomania with mixed features	0 (0%)	1 (2.1%)	1 (1.6%)
Depressive with mixed features	0 (0%)	3 (6.4%)	3 (4.8%)
**Psychiatric comorbidities** (yes/no)	11 (69.8%); 5 (31.3%)	26 (50%); 26 (50%)	37 (54.4%); 31 (45.6%)
**Lifetime Psychiatric comorbidities**			
None	5 (31.1%)	26 (50%)	31 (45.6%)
Generalized Anxiety Disorder	2 (12.5%)	8 (15.4%)	10 (14.7%)
Panic Disorder	0 (0%)	5 (9.6%)	5 (7.4%)
Obsessive-compulsive Disorder	2 (12.5%)	0 (0%)	2 (2.9%)
Personality Disorder	0 (0%) **	**4 (7.7%)**	4 (5.9%)
Alcohol/Substance Use disorder	6 (37.5%) **	**5 (9.6%)**	11 (16.2%)
Eating disorder	0 (0%)	4 (7.7%)	4 (5.9%)
Post-Traumatic Stress Disorder	1 (6.3%)	0 (0%)	1 (1.5%)
**Medical comorbidities** (yes/no)	8 (42.1%); 11 (57.9%)	35 (66%); 18 (34%)	43 (59.7%); 29 (40.3%)
**Type of Medical comorbidities**			
None	14 (58.3%)	20 (33.3%)	34 (40.5%)
Celiac disease	0 (0%)	1 (1.7%)	1 (1.2%)
Hypertension	7 (29.2%)	9 (15%)	16 (19%)
Any headache disorders	0 (0%)	4 (6.7%)	4 (4.8%)
Thyroid disease	0 (0%)	6 (10%)	6 (7.1%)
Hypercholesterolemia	1 (4.2%)	2 (3.3%)	3 (3.6%)
Neurodegenerative disorders	0 (0%)	3 (7.1%)	3 (4.8%)
Other	2 (8.3%)	14 (23.3%)	16 (19%)
**Current substance use** (yes/no)	3 (14.3%); 18 (85.7%)	2 (3.9%); 49 (96.1%)	5 (6.9%); 67 (93.1%)
**N° of Hospitalizations**			
None	2 (11.1%) *	**25 (46.3%)**	27 (37.5%)
From 1 to 3	4 (22.2%)	14 (25.9%)	18 (25%)
From 3 to 6	6 (33.3%)	9 (16.7%)	15 (20.8%)
From 7 to 10	4 (22.2%)	4 (7.4%)	8 (11.1%)
Over 10	2 (11.1%)	2 (3.7%)	4 (5.6%)
**N° of Involuntary commitments**			
None	10 (62.5%) *	**49 (90.7%)**	59 (84.3%)
From 1 to 3	3 (18.8%)	5 (9.3%)	8 (11.4%)
From 4 to 6	2 (12.5%) *	**0 (0%)**	2 (2.9%)
Over 10	1 (6.3%)	0 (0%)	1 (1.4%)
**Psychotherapy lifetime** (yes/no)	6 (23.1%); 20 (76.9%)	23 (41.1%); 33 (58.9%)	29 (35.4%); 53 (64.6%)

**Notes:** Values for categorical and continuous variables are expressed in percentages and mean ± SD, respectively. A+: positive adherence to treatment, A−: negative adherence to treatment, DUI: duration of untreated illness. Reported variables had a percentage of missing data ranging from 0% to 14%. Boldface indicates parameters with statistically significant differences between subgroups. ** *p* < 0.005 * *p* < 0.05.

**Table 2:** Comparison of psychometric questionnaires between subgroups.

Psychometric Questionnaires	A−	A+	Total Sample
CRS (mean, sd)	2.59 ± 1.04	5.77 ± .69	4.70 ± 1.72 (N = 108)
HAM-A (mean, sd)	5.63 ± 13.6 *	**12.9 ± 18.3**	12 ± 6.1 (N = 102)
HDRS-21 (mean, sd)	6.8 ± 17.8	14.3 ± 20.3	13.7 ± 18.8 (N = 102)
MADRS (mean, sd)	5.6 ± 25.3	19.7 ± 27.7	18.3 ± 25.7 (N = 102)
MoCa (mean, sd)	20.5 ± 13.8	20.9 ± 11.8	20.8 ± 11.8 (N = 90)

**Notes:** Values for continuous variables are expressed in percentages and mean ± SD, respectively. SD: standard deviation; A+: positive adherence to treatment, A−: negative adherence to treatment, CRS: Clinician Rating Scale, HAM-A: Hamilton Anxiety Rating Scale, HDRS-21: Hamilton Depression Rating Scale, MADRS: Montgomery Asberg Depression Rating Scale, MoCa: Montreal Cognitive Assessment. Reported variables had a percentage of missing data ranging from 0% to 14%. Boldface indicates parameters with statistically significant differences between subgroups. * *p* < 0.05.

## Data Availability

Data are available upon request to the corresponding author.
